# Evaluating Outcomes for Women with Metastatic Breast Cancer: Palliative Care Consultations, Hospital Charges, and Length of Stay

**DOI:** 10.3390/cancers16223724

**Published:** 2024-11-05

**Authors:** Leslie J. Hinyard, Divya S. Subramaniam, Alexandria M. Jenkins, Zachary Timmer, Noor Al-Hammadi

**Affiliations:** 1 Department of Health and Clinical Outcomes Research, School of Medicine, Saint Louis University, St. Louis, MO 63104, USA; 2 Advanced HEAlth Data (AHEAD) Institute, Saint Louis University, St. Louis, MO 63104, USA; 3 School of Medicine, Saint Louis University, St. Louis, MO 63104, USA

**Keywords:** Palliative care, metastatic breast cancer, inpatient, length of stay, hospitalization charges

## Abstract

**Introduction**: Women with late-stage metastatic breast cancer are at an increased risk of pain and distress from symptoms and often struggle with associated emotional and financial burden of their disease. Palliative care is known to alleviate symptom burden in patients with end-stage, terminal diseases but is often underutilized in both inpatient and outpatient settings. The current study aims to investigate the prevalence of palliative care consultation on inpatients with metastatic breast cancer and examine the association between palliative care consultation and length of hospital stay and total hospital charges. **Methods**: Patients diagnosed with metastatic breast cancer between 1998–2017 were abstracted from the Healthcare Cost and Utilization Project (HCUP) Nationwide Inpatient Database (NIS). The primary outcome was the presence of a palliative care consultation (PCC) during the inpatient stay. Secondary outcomes were hospital length of stay and total hospital charges. Multivariable logistic regression was used to examine factors associated with the presence of a PCC. The relationship between PCC and hospital length of stay and total hospital charges were investigated using linear regression. **Results**: 513,509 cases of metastatic breast cancer were identified, 5.7% had a documented in-hospital palliative care encounter. Of those who received PCC, total hospital charges were about USD 5452 less than those who did not receive consultation. Women who received PCC had higher odds of a longer hospital stay. Predictors of PCC were older age, non-White race, and residing in a lower-income ZIP code. **Conclusions**: Palliative care remains to be an underutilized resource among patients with end-stage metastatic breast cancer.

## Simple Summary

Metastatic breast cancer (MBC) frequently results in increased pain and distress for women affected by the disease. Management of these symptoms can be complicated and expensive, leading to undue stress for these patients and their families. Palliative care has demonstrated effectiveness in alleviating symptomology due to disease and improving quality of life for patients with MBC, yet utilization of palliative care remains low. The purpose of this study is to document the prevalence of palliative care consultations among inpatients with metastatic breast cancer and to examine the impact of palliative care on the length of hospital stay and total hospital charges. The results of this study demonstrate that only 5.7% of patients with metastatic breast cancer received a palliative care consultation during their inpatient stay. Palliative care consultation was associated with a longer length of stay but lower hospital charges.

## 1. Introduction

Breast cancer is the most commonly diagnosed malignancy worldwide [1]. In the United States, approximately 6% of women with incident breast cancer will be diagnosed with metastatic disease [2]. There are currently no known estimates of the number of women diagnosed with earlier stage breast cancer that go on to develop metastatic disease [2,3]. It is well documented that women with late-stage, metastatic breast cancer are at risk of untreated pain and distress from other symptoms [4,5,6], struggle with emotional and financial burdens of their disease [7,8,9], and may have poor communication in their conversations with physicians and other providers [10].

Palliative intervention has been shown to significantly improve patient outcomes over multiple measures, including quality of life, cost of care, and healthcare utilization of aggressive treatments at the end of life. Notably, the use of palliative care in patients with advanced cancers has been especially promising in these areas [11,12,13]. Patients with a terminal cancer diagnosis tend to live longer and experience higher rates of comorbidities compared to other terminal conditions [12,14], indicating a potentially longer-term need for palliation compared to other terminal conditions. Additionally, the rising costs of cancer therapies and pharmaceuticals present economic challenges, disproportionately affecting those with cancer over patients with other chronic illnesses [15]. For cancer patients, early palliative care and palliative care in inpatient settings have demonstrated reductions in aggressive treatment at the end-of-life [16]; reduction in costs at end-of-life—two studies demonstrated an average cost reduction of USD 3823 for those with PCC [12,17]; and increased likelihood of patient dying in settings outside of the hospital [18], though in some instances, an increase in inpatient hospital mortality has been documented [12].

Although the benefits of palliative care for cancer patients are widely documented, low rates of referral and end-of-life conversations may contribute to lack of utilization and knowledge of advanced care planning options [14]. Despite recognized benefits of palliative care for patients with cancer, there is limited research about the actual utilization of palliative care services for women with metastatic breast cancer, particularly at end-of-life. The purpose of this study was to determine the incidence of inpatient palliative care consultations in women with metastatic breast cancer and to examine the association between palliative care consultation and hospital length of stay and in-hospital charges in a nationally representative sample.

## 2. Materials and Methods

### 2.1. Data Source and Sample Selection

This study is a retrospective analysis of the 1998–2017 Healthcare Cost and Utilization Project (HCUP) Nationwide Inpatient Database (NIS, https://hcup-us.ahrq.gov/db/nation/nis/nisdbdocumentation.jsp, Accessed 16 June 2020), which utilizes an approximated, self-weighted sample of discharges from all-payer billing data submitted to statewide data organizations from participating hospitals in 47 states of the United States and the District of Columbia [19,20], including academic and community hospitals but excluding rehabilitation centers and long-term acute care facilities [20]. Metastatic-breast-cancer-related hospitalizations of women ages 18 years and older were identified using a combination of the International Classification of Diseases Clinical Modification versions 9 and 10 (Appendix A. Table A1).

### 2.2. Ethical Considerations

Saint Louis University’s Institutional Review Board deemed this to be non-human subject research.

### 2.3. Outcomes Variables

The primary outcome for this analysis was the presence of a palliative care consultation (PCC) during inpatient hospitalization as identified by ICD-9 (V66.7) and ICD-10 (Z51.5) codes. The ICD-9 code (V66.7) for PCC has been validated by multiple studies within the United States for patients with chronic, life-threatening illnesses. These studies found the code to be highly specific (>90%) and moderately sensitive (45–89%) [21]. The ICD-10 code (Z51.5) needs further validation but is grossly equivalent to V66.7 [22]. Total charges associated with hospitalization and length of hospital stay (LOS) were also evaluated as secondary outcomes. The HCUP database total charge variable directly represents the amount a hospital billed for a case reported by inpatient encounter records [23]. Total charges were inflation-adjusted to 2011 dollars using the Consumer Price Index (CPI) from the US Bureau of Labor Statistics [24].

### 2.4. Predictor Variables

Patient characteristics included age, sex, race, annual median household income of patient county of residence, and health insurance coverage. Hospital characteristics of interest were bed size, region within the US, urban/rural location, and teaching status of the hospital. Geographic regions (Northeast, South, Midwest, and West) were defined according to the Census Bureau [25]. For descriptive stratification, age was categorized as <40, 40–49, 50–59, 60–69, and 70+ years of age. Race was categorized as White, Black, and other races. Insurance status was classified as private, government (Medicare or Medicaid), or other insurance. Comorbid risk factors for PCC were identified from the literature and defined using ICD-9/10 diagnosis codes and were classified as present or absent. Comorbid risk factors in this analysis included sepsis, weight loss, acute renal failure, pneumonia, dehydration, urinary tract infections (UTI), acute cardiac event, acute pulmonary event, acute cerebrovascular event, and acute hepatic failure.

### 2.5. Statistical Considerations

Summary statistics are presented as frequencies and proportions to examine the total inpatient metastatic breast cancer burden and stratified by the presence or absence of PCC. The distribution of patient sociodemographic factors, comorbidities, and hospital characteristics were evaluated as bivariate associations using the Rao–Scott chi-square test. Student’s t-tests were calculated to examine the differences in mean of each of the secondary outcomes including length of stay and total hospital charges across PCC status for all patients in the study sample and within each comorbidity subpopulation.

Multivariable logistic regression analysis for the primary outcome (PCC) and the secondary outcome (LOS categorized as a bivariate variable at the median of 4 days) and linear regression analysis evaluating predictors of inflation-adjusted hospital charges were performed to adjust for the baseline patient and hospital characteristics and patient comorbidities. The charge outcome variable was skewed; however, we did not transform the variable as parametric methods are robust to nonnormality in small samples [26]. Variables in the models were selected based on prior literature. Discharge weights were used to obtain national estimates at the visit level. All odds ratios (ORs) and beta coefficients are presented with 95% confidence intervals and statistical significance was determined at the alpha level of 0.05. SAS software survey procedures (version 9.4, SAS Institute Inc., Cary, NC, USA) were used for all analyses to account for the sampling design of NIS.

## 3. Results

There were 513,509 cases in 1998–2017 that met the study inclusion criteria. Table 1 presents the patient and hospital characteristics of the study sample. Among the study sample, 5.7% had an in-hospital palliative care encounter (PCC). In-hospital mortality was 7.6%. The average age was 63 years old, and most patients were White (73.2%), on government insurance (58.4%), and lived in ZIP codes with annual household incomes above the 50th percentile (25.5% in 51st–75th percentiles, 31.5% in 71st–100th percentile).

In bivariate analysis (Table 1), PCC was more likely in women who were over 70 years (37.4%), White (68.5%), and on government insurance (59.8%). Death during hospitalization was more common among women who had PCC compared to those who did not (34.4% versus 5.9%, respectively; *p* < 0.001).

Table 2 presents predictors of PCC. Advanced age was associated with higher odds of PCC (aOR, 95% CI (60–69 years: 1.08 (1.01, 1.16); ≥70 years: 1.25 (1.17, 1.34))). Furthermore, women of races other than White (Black: 1.12 (1.07, 1.16); other races 1.08 (1.04, 1.13)), and patients from ZIP codes with lower household income levels were more likely to receive a palliative care consult (0–25th percentile: 1.17 (1.12, 1.21); 26–50th percentile 1.05 (1.01, 1.09); 51–75th percentile 1.06 (1.02, 1.20)). Insurance status was also associated with receipt of PCC, where women with “other” insurance were more likely to receive a PCC compared to those with private insurance (1.97 (1.86, 2.09)), while women reporting government insurance had slightly lower odds of receiving PCC compared to women receiving commercial types of insurance [0.94 (0.91, 0.98)]. Smaller hospital size, rural hospitals, and non-teaching hospitals were also associated with lower PCC (Table 2). All examined comorbid conditions were significant predictors of PCC (Table 2).

In linear regression analysis, total charges associated with hospitalization for patients receiving PCC were about USD 5452 (95% CI: −5968, −4936) less than women without a PCC encounter (Table 2). Lower charges were also associated with women older than 40, and those with non-private insurance coverage. Alternatively, higher charges were seen in patients with longer LOS and in other races when compared to White patients. Patients admitted with any of the comorbidities of interest in this study, except dehydration, were likely to have higher charges at discharge (Table 2).

Women who received PCC were more likely to have an in-hospital LOS > 4 days compared to those without a PCC encounter (1.17 (1.14, 1.20)). Women older than 40 years, non-White patients and patients receiving non-private insurances were also more likely to have a LOS longer than 4 days. Small- and medium-bed-size hospitals, rural and non-teaching hospitals were associated with shorter hospital stays (Table 2).

Incidence of comorbid conditions among all MBC patients and those with PCC are detailed in Table A2. Mean LOS and total charge for each comorbidity of interest overall and stratified by the receipt of PCC are shown in Table 3 and Table 4. There were significant differences in the mean LOS for those who received PCC as compared to those who did not in patients who had an associated diagnosis code of sepsis, weight loss, acute renal failure, pneumonia, dehydration, acute pulmonary event, and acute hepatic failure. For total charges, these significant differences were noted in women who had sepsis, weight loss, pneumonia, dehydration, urinary tract infections and acute cardiac events.

## 4. Discussion

Palliative care has demonstrated benefits in patients with cancer including improvement in quality of life at the end of life and lower associated costs [10,14,15,18]. Despite established benefits, there is insufficient research investigating the use of inpatient palliative care consultations among women diagnosed with metastatic breast cancer. Given the significant disease burden for metastatic breast cancer patients [2,3,4,5], this study aimed to understand the association between palliative care consultation, hospital length of stay, and in-hospital-related charges. We found that only 5.7% of inpatients with metastatic breast cancer received a palliative care consultation during their hospitalization, despite existing evidence that women with metastatic breast cancer experience a higher burden of psychological distress and pain prior to the end of life [4]. In other words, 94.3% of patients with metastatic breast cancer did not receive an inpatient palliative care consultation. While the rate of PCC in metastatic breast cancer is comparable to what has been described in other metastatic cancers [12,27,28,29], the low utilization of PCC represents a missed opportunity for reductions in patient suffering, improvements in end-of-life care, and reduction in cost of care at the end of life.

Aligned with findings in other metastatic cancers [12,27,28], we found that metastatic breast cancer patients were more likely to have an inpatient PCC if they were not White. After controlling for other covariates, we found that Black women were 20% more likely to have an inpatient PCC compared to White women, and women of other races were 11% more likely to have a PCC. The increased incidence of PCC in Black patients compared to White patients is consistent across the palliative care literature in both oncology [12,27,28,30] and non-oncology [31] settings. One plausible explanation is that inpatient palliative care services are only offered to the sickest patients and that non-White patients are more likely to be sicker during their hospitalization than White patients. This assertion is supported by our findings that patients were more likely to have PCC if they had acute comorbid conditions. Another possibility is that non-White patients may be perceived as having less social or financial support than their White counterparts and, thus, providers are more likely to refer to additional support services, including palliative care.

Consistent with literature [12,17,29], we found that patients who received PCC incurred lower charges overall when compared to patients who did not receive any PCC during the study period. After adjusting for our secondary outcomes, (1) hospital length of stay and (2) associated charges, we found that non-white patients and patients admitted with any of the comorbidities of interest, except dehydration, were likely to have higher charges. It is probable that non-white patients are sicker upon referral or are more likely to have comorbidities that increase overall charges. There is evidence that PCC may not correlate with decreased health care costs among Black patients to the same extent seen in White patients [32]. This warrants that more research is needed to better understand the underlying cause of these existing racial inequities in service utilization for those with advanced metastatic diseases.

Furthermore, we also found that older patients, patients with non-private insurance, and those treated in smaller and medium size hospitals were likely to have lower medical charges. Though we might expect that older patients may have other comorbidities that could lead to increased costs [20] the lower charges associated with women over 40 may be due to less demanding medical intervention with increased age. A study by Mulvey et al. (2016) utilized a similar study design to the current study and found that in a cohort of patients with metastatic head and neck cancer, Medicaid insurance was associated with higher hospital costs while Medicare and self-pay/other had no difference compared to private insurance [12]. In the current study, we found that any government insurance was associated with longer LOS but reduced overall hospital charges compared to those with commercial insurance. While these results appear inconsistent, we were unable to separate Medicaid and Medicare in the current study. It is possible that, given the demonstrated differential effects of median income in ZIP code of reference, the combination of Medicare and Medicaid into one overall group is masking the real relationship between insurance status and hospital charges. More research on the differences in hospital costs based on insurance status for end-of-life care could address these discrepancies and provide insight into barriers correlated with palliative care utilization.

Finally, larger hospitals may have higher associated costs due to the challenges of maintaining a larger hospital and a more complex and diverse patient base. Though there is supporting evidence that larger hospitals are associated with higher costs at the end of life [12], more research on palliative care and its potential cost-saving benefits may be especially helpful in decreasing this cost difference at larger institutions. Academic institutions, while typically thought of as more expensive than non-teaching hospitals, have recent contradictory evidence indicating that there may be no difference in cost [12,33]. Nevertheless, more research is needed to further understand the types of institutions and their impact on associated costs.

As with any study, it is important to acknowledge limitations. There is likely some misclassification of PCC within the context of this study; however, there is no indication this misclassification is differential. Furthermore, the NIS only provides a cross-sectional view. It is likely that some patients within the cohort had a PCC encounter at an earlier hospitalization or were receiving outpatient palliative care services; thus, negating the need for an inpatient consultation. Lastly, the HCUP database does not include specific hospital identifiers, making it impossible to determine which hospitals have access to palliative care services. Despite these limitations, there were several notable strengths with this study. First, the NIS is a nationally representative, inpatient, all payer, publicly available database in the United States. For that reason, a major strength of this study is that these findings can be generalized to the metastatic breast cancer population within the United States. Second, to our best knowledge, this is one of the largest studies to assess disparities in palliative care consultation, hospital length of stay, and in-hospital related charges among metastatic breast cancer patients. Lastly, the use of the ICD 9/10 codes V66.7 and Z51.5, respectively, as the method to determine the utilization of palliative care consultation among patients in the United States with critical, life-threatening illnesses, has been demonstrated by previous studies to be a specific method [21,22].

## 5. Conclusions

We found that inpatient PCC for women with metastatic breast cancer was exceptionally low given the severity of disease. Additionally, PCC was associated with increased LOS and decreased hospital charges. Palliative care is often plugged as a mechanism for decreasing overall hospitalizations and cost of care [11,12,16,17]. We also found that inpatient PCC was provided to sicker patients—patients who would likely have longer LOS and higher hospital costs compared to those without multiple acute comorbid conditions. Future research needs to carefully control the acuity of patient illnesses, something not possible with this data source. Additionally, the quality of palliative care consult as well as the receipt of palliative care services should be examined to understand how receipt of services, rather than simply consultation for services, effect outcomes in women with metastatic breast cancer. Future work should also examine how more equitable distribution of palliative care services influences patient and system outcomes. Lastly, future research and policies must aim to develop targeted strategies and interventions to increase palliative care consultation and utilization to decrease existing disparities among metastatic breast cancer patients.

## Figures and Tables

**Table 1:** Demographic and hospital characteristics of metastatic breast cancer patients stratified by receipt of palliative care consultation.

		Palliative Care Consultation (PCC)		
	Full Sample	Yes	No	*p*-Value *
	N (%)	N (%)	N (%)	
Patient Characteristics				
Age (mean, std err)	62.7 (0.02)	64.2 (0.08)	62.6 (0.02)	<0.0001
<40	27,313 (5.3)	1372 (4.7)	25,941 (5.4)	
40–49	73,772 (14.4)	3416 (11.7)	70,356 (14.5)	
50–59	115,672 (22.5)	6463 (22.1)	109,209 (22.5)	
60–69	121,776 (23.7)	7055 (24.1)	114,721 (23.7)	
70+	175,076 (34.1)	10,945 (37.4)	164,131 (33.9)	
Race				<0.0001
White	314,465 (73.2)	18,432 (68.5)	296,033 (73.50	
Black	62,632 (14.6)	4817 (17.9)	57,815 (14.4)	
Other	52,640 (12.2)	3639 (13.5)	49,001 (12.1)	
Missing	83,872	2363	81,509	
Insurance Payer				<0.0001
GOV ^a^	299,264 (58.4)	17,458 (59.8)	281,806 (58.3)	
Private	191,759 (37.4)	9625 (33.0)	182,134 (37.7)	
Other	21,675 (4.2)	2098 (7.2)	19,577 (4.0)	
Missing	911	70	841	
Median household income category for patient’s ZIP code				<0.0001
0–25th percentile	95,910 (19.0)	6620 (23.1)	89,290 (18.8)	
26th to 50th percentile (median)	121,113 (24.1)	6683 (23.3)	114,430 (24.1)	
51st to 75th percentile	128,060 (25.5)	7252 (25.3)	120,808 (25.5)	
76th to 100th percentile	158,011 (31.4)	8122 (28.3)	149,889 (31.6)	
Missing	10,515	574	9941	
Died during hospitalization				<0.0001
Yes	38,771 (7.6)	10,060 (34.4)	28,711 (5.9)	
No	474,325 (92.4)	19,164 (65.6)	455,161 (94.1)	
Missing	513	27	486	
Hospital Characteristics				
Bed size of hospital				<0.0001
Small	63,322 (12.0)	3377 (11.4)	59,945 (12.0)	
Medium	122,509 (23.8)	7262 (25.0)	115,247 (23.7)	
Large	326,596 (64.2)	18,530 (63.7)	308,066 (64.3)	
Missing	1182	82	1100	
Location/teaching status of hospital				<0.0001
Rural	49,163 (9.7)	1959 (6.8)	47,204 (9.9)	
Urban nonteaching	189,397 (36.3)	8734 (29.5)	180,663 (36.7)	
Urban teaching	273,867 (54.0)	18,476 (63.7)	255,391 (53.4)	
Missing	1182	82	1100	
Region of hospital				<0.0001
Northeast	118,178 (23.7)	6208 (21.6)	111,970 (23.8)	
Midwest	115,109 (22.8)	6431 (22.1)	108,678 (22.9)	
South	181,400 (34.6)	10,378 (35.2)	171,022 (34.5)	
West	98,922 (18.9)	6234 (21.1)	92,688 (18.8)	
Total Charges (mean, std)		51,608 (461.8)	43,330 (79.4)	<0.0001
Length of hospital stay (mean, std)		7 (0.04)	6 (0.01)	<0.0001

* Chi-square test. ^a^ Gov, government (Medicare or Medicaid).

**Table 2:** Predictors of length of hospital stay and total charges of patients receiving PCC Compared to the non-PCC group.

	Have at Least 1 Encounter of PCC ^a^	LOS ^b^ (>4 Days)	Inflation-Adjusted Total Charges, R-sq = 44.93%
	aOR (95% CI)	aOR	Adjusted Estimate
Palliative Care Encounter (PCC)		1.167 (1.135, 1.200)	−5452 (−5968, −4936)
Length of hospital stay (>4 days)			5399 (5241, 5557)
Patient Characteristics			
Age			
<40 (reference)			
40–49	0.936 (0.873, 1.004)	1.092 (1.057, 1.129)	−1448 (−2053, −833)
50–59	1.062 (0.995, 1.134)	1.278 (1.239, 1.319)	−2805 (−3392, −2218)
60–69	1.082 (1.013, 1.157)	1.331 (1.290, 1.373)	−3896 (−4482, −3310)
70+	1.253 (1.169, 1.342)	1.424 (1.379, 1.470)	−8365 (−8965, −7766)
Race			
White (reference)			
Black	1.115 (1.074, 1.158)	1.234 (1.211, 1.258)	16 (−364, 395)
Other	1.080 (1.037, 1.125)	1.040 (1.019, 1.062)	5337 (4936, 5739)
Insurance Payer			
Private (reference)			
GOV ^c^	0.944 (0.910, 0.979)	1.226 (1.207, 1.246)	−2236 (−2541, −1930)
Other	1.970 (1.861, 2.085)	1.043 (1.009, 1.079)	−4807 (−5336, −4277)
Median household income category for patient’s zip code			
0–25th percentile	1.166 (1.120, 1.213)	0.976 (0.957, 0.995)	1830 (1471, 2188)
26th to 50th percentile (median)	1.051 (1.013, 1.091)	1.001 (0.983, 1.019)	−1020 (−1337, −702)
51st to 75th percentile	1.058 (1.021, 1.097)	0.996 (0.979, 1.013)	4 (−311, 318)
76th to 100th percentile (reference)			
Hospital Characteristics			
Bed size of hospital			
Small	0.873 (0.838, 0.910)	0.717 (0.703, 0.732)	−6273 (−6636, −5910)
Medium	0.995 (0.965, 1.026)	0.885 (0.871, 0.898)	−3657 (−3914, −3399)
Large (reference)			
Location/teaching status of hospital			
Rural	0.619 (0.586, 0.654)	0.759 (0.741, 0.778)	−17,032 (−17,376, −16,688)
Urban nonteaching	0.663 (0.644, 0.682)	0.994 (0.980, 1.008)	−4085 (−4343, −3827)
Urban teaching (reference)			
Region of hospital			
Northeast	0.774 (0.744, 0.805)	1.428 (1.401, 1.456)	−19,793 (−20,233, −19,353)
Midwest	0.889 (0.852, 0.928)	1.199 (1.173, 1.226)	−25,738 (−26,136, −25,339)
South	0.848 (0.818, 0.879)	1.243 (1.221, 1.266)	−23,277 (−23,636, −22,917)
West (reference)			
Comorbidities			
Sepsis	2.490 (2.291, 2.707)	1.377 (1.287, 1.474)	12,087 (9849, 14,326)
Weight loss	2.821 (2.727, 2.918)	2.003 (1.956, 2.050)	4220 (3607, 4832)
Acute renal failure	1.993 (1.913, 2.077)	1.605 (1.560, 1.650)	13,278 (12,574, 13,981)
Pneumonia	1.315 (1.232, 1.403)	2.197 (2.103, 2.296)	19,091 (17,539, 20,643)
Dehydration	1.547 (1.483, 1.615)	0.934 (0.909, 0.959)	−3707 (−4108, −3306)
UTI ^d^	1.138 (1.094, 1.185)	2.015 (1.970, 2.060)	76 (−483, 635)
Acute cardiac event	1.064 (1.020, 1.110)	1.448 (1.416, 1.482)	1610 (1101, 2119)
Acute pulmonary event	2.723 (2.603, 2.848)	1.839 (1.782, 1.899)	25,769 (24,774, 26,764)
Acute cerebrovascular event	1.628 (1.180, 2.245)	3.981 (3.217, 4.926)	42,816 (34,956, 50,676)
Acute hepatic failure	3.093 (2.751, 3.476)	0.792 (0.719, 0.874)	7268 (4717, 9819)

^a^ PCC, palliative care consultation. ^b^ LOS, length of stay. ^c^ GOV, government (Medicare or Medicaid). ^d^ UTI, urinary tract infection.

**Table 3:** Length of hospital stay and total charges for All MBC patients.

Comorbidities	All Patients	
	LOS ^a^	Total Charges
	Mean, Std Err ^b^	Mean, Std Err
Sepsis	8 (0.12)	80,368 (1946.1)
Weight Loss	8 (0.04)	63,877 (416.1)
Acute Renal Failure	8 (0.05)	72,702 (577.0)
Pneumonia	10 (0.09)	94,781 (1175.2)
Dehydration	6 (0.03)	43,876 (296.8)
UTI ^c^	8 (0.04)	59,213 (352.3)
Acute Cardiac Event	7 (0.04)	54,616 (352.3)
Acute Pulmonary Event	9 (0.07)	91,546 (775.3)
Acute Cerebrovascular Event	11 (0.43)	115,661 (6238.0)
Acute Hepatic Failure	7 (0.14)	67,159 (1918.3)

^a^ LOS, length of stay. ^b^ std err, standard error. ^c^ UTI, urinary tract infection.

**Table 4:** Comparison of length of hospital stay and total charges between PCC and non-PCC groups.

Comorbidities	PCC ^a^ (Yes)		PCC (No)		PCC Comparisons	
	LOS ^b^	Total Charges	LOS	Total Charges	*p*-Value	
	Mean, Std Err ^c^	Mean, Std Err	Mean, Std Err	Mean, Std Err	LOS	Total Charges
Sepsis	7 (0.22)	85.809 (4895.8)	8 (0.14)	79.248 (2147.7)	<0.0001	0.2276
Weight Loss	8 (0.10)	64.830 (1218.2)	8 (0.04)	63.708 (445.7)	0.0007	0.3903
Acute Renal Failure	7 (0.11)	72.917 (1562.3)	8 (0.05)	72.664 (627.2)	<0.0001	0.8809
Pneumonia	9 (0.23)	100.711 (3511.1)	10 (0.10)	93.966 (1260.9)	0.0007	0.0728
Dehydration	7 (0.10)	50.024 (1099.5)	6 (0.03)	43.133 (309.0)	<0.0001	<0.0001
UTI ^d^	9 (0.15)	69.365 (1513.3)	8 (0.05)	58.353 (363.1)	0.0622	<0.0001
Acute Cardiac Event	7 (0.14)	65.079 (1650.5)	7 (0.04)	53.830 (361.0)	0.1108	<0.0001
Acute Pulmonary Event	8 (0.13)	85.196 (1737.0)	10 (0.07)	92.842 (873.2)	<0.0001	0.0001
Acute Cerebrovascular Event	11 (1.16)	122.879 (18.712.0)	11 (0.46)	115.067 (6620.1)	0.7478	0.7345
Acute Hepatic Failure	6 (0.23)	61.192 (3050.8)	7 (0.17)	69.298 (2409.5)	0.0001	0.0401

^a^ PCC, palliative care consultation. ^b^ LOS, length of stay. ^c^ std err, standard error. ^d^ UTI, urinary tract infection.

## Data Availability

Data are available through the Agency for Healthcare Research and Quality (AHRQ).

## Appendix A

**Table A1:** ICD-9 and ICD-10 Codes for diagnosis of breast cancer and metastatic classification.

**ICD-9**	**Cancer of the Breast**
174	Malignant neoplasm of nipple and areola of female breast
174.1	Malignant neoplasm of central portion of female breast
174.2	Malignant neoplasm of upper-inner quadrant of female breast
174.3	Malignant neoplasm of lower-inner quadrant of female breast
174.4	Malignant neoplasm of upper-outer quadrant of female breast
174.5	Malignant neoplasm of lower-outer quadrant of female breast
174.6	Malignant neoplasm of axillary tail of female breast
174.8	Malignant neoplasm of other specified sites of female breast
174.9	Malignant neoplasm of breast (female), unspecified
233	Carcinoma in situ of breast
V103	Personal history of malignant neoplasm of breast
**ICD-10**	**Cancer of the Breast**
C50.011	Malignant neoplasm of nipple and areola, right female breast
C50.012	Malignant neoplasm of nipple and areola, left female breast
C50.019	Malignant neoplasm of nipple and areola, unspecified female breast
C50.111	Malignant neoplasm of central portion of right female breast
C50.112	Malignant neoplasm of central portion of left female breast
C50.119	Malignant neoplasm of central portion of unspecified female breast
C50.211	Malignant neoplasm of upper-inner quadrant of right female breast
C50.212	Malignant neoplasm of upper-inner quadrant of left female breast
C50.219	Malignant neoplasm of upper-inner quadrant of unspecified female
C50.311	Malignant neoplasm of lower-inner quadrant of right female breast
C50.312	Malignant neoplasm of lower-inner quadrant of left female breast
C50.319	Malignant neoplasm of lower-inner quadrant of unspecified female
C50.411	Malignant neoplasm of upper-outer quadrant of right female breast
C50.412	Malignant neoplasm of upper-outer quadrant of left female breast
C50.419	Malignant neoplasm of upper-outer quadrant of unspecified female
C50.511	Malignant neoplasm of lower-outer quadrant of right female breast
C50.512	Malignant neoplasm of lower-outer quadrant of left female breast
C50.519	Malignant neoplasm of lower-outer quadrant of unspecified female
C50.611	Malignant neoplasm of axillary tail of right female breast
C50.612	Malignant neoplasm of axillary tail of left female breast
C50.619	Malignant neoplasm of axillary tail of unspecified female breast
C50.811	Malignant neoplasm of overlapping sites of right female breast
C50.812	Malignant neoplasm of overlapping sites of left female breast
C50.819	Malignant neoplasm of overlapping sites of unspecified female breast
C50.911	Malignant neoplasm of unspecified site of right female breast
C50.912	Malignant neoplasm of unspecified site of left female breast
C50.919	Malignant neoplasm of unspecified site of unspecified female breast
D05.00	Lobular carcinoma in situ of unspecified breast
D05.01	Lobular carcinoma in situ of right breast
D05.02	Lobular carcinoma in situ of left breast
D05.80	Other specified type of carcinoma in situ of unspecified breast
D05.81	Other specified type of carcinoma in situ of right breast
D05.82	Other specified type of carcinoma in situ of left breast
D05.90	Unspecified type of carcinoma in situ of unspecified breast
D05.91	Unspecified type of carcinoma in situ of right breast
D05.92	Unspecified type of carcinoma in situ of left breast
**ICD-9**	**Metastatic Classification**
196	Secondary and unspecified malignant neoplasm of lymph nodes of head, face, and neck
196.1	Secondary and unspecified malignant neoplasm of intrathoracic lymph nodes
196.2	Secondary and unspecified malignant neoplasm of intra-abdominal lymph nodes
196.3	Secondary and unspecified malignant neoplasm of lymph nodes of axilla and upper limb
196.5	Secondary and unspecified malignant neoplasm of lymph nodes of inguinal region and lower limb
196.6	Secondary and unspecified malignant neoplasm of intrapelvic lymph nodes
196.8	Secondary and unspecified malignant neoplasm of lymph nodes of multiple sites
196.9	Secondary and unspecified malignant neoplasm of lymph nodes, site unspecified
197	Secondary malignant neoplasm of lung
197.1	Secondary malignant neoplasm of mediastinum
197.2	Secondary malignant neoplasm of pleura
197.3	Secondary malignant neoplasm of other respiratory organs
197.4	Secondary malignant neoplasm of small intestine including duodenum
197.5	Secondary malignant neoplasm of large intestine and rectum
197.6	Secondary malignant neoplasm of retroperitoneum and peritoneum
197.7	Malignant neoplasm of liver, secondary
197.8	Secondary malignant neoplasm of other digestive organs and spleen
198	Secondary malignant neoplasm of kidney
198.1	Secondary malignant neoplasm of other urinary organs
198.2	Secondary malignant neoplasm of skin
198.3	Secondary malignant neoplasm of brain and spinal cord
198.4	Secondary malignant neoplasm of other parts of nervous system
198.5	Secondary malignant neoplasm of bone and bone marrow
198.6	Secondary malignant neoplasm of ovary
198.7	Secondary malignant neoplasm of adrenal gland
198.8	Secondary malignant neoplasm of other specified sites
**ICD-10**	**Metastatic Classification**
C77.0	Secondary and unspecified malignant neoplasm of lymph nodes of head, face and neck
C77.1	Secondary and unspecified malignant neoplasm of intrathoracic lymph nodes
C77.2	Secondary and unspecified malignant neoplasm of intra-abdominal lymph nodes
C77.3	Secondary and unspecified malignant neoplasm of axilla and upper limb lymph nodes
C77.4	Secondary and unspecified malignant neoplasm of inguinal and lower limb lymph nodes
C77.5	Secondary and unspecified malignant neoplasm of intrapelvic lymph nodes
C77.8	Secondary and unspecified malignant neoplasm of lymph nodes of multiple regions
C77.9	Secondary and unspecified malignant neoplasm of lymph node, unspecified
C78.0	Secondary malignant neoplasm of lung
C78.00	Secondary malignant neoplasm of unspecified lung
C78.01	Secondary malignant neoplasm of right lung
C78.02	Secondary malignant neoplasm of left lung
C78.1	Secondary malignant neoplasm of mediastinum
C78.2	Secondary malignant neoplasm of pleura
C78.3	Secondary malignant neoplasm of other and unspecified respiratory organs
C78.30	Secondary malignant neoplasm of unspecified respiratory organ
C78.39	Secondary malignant neoplasm of other respiratory organs
C78.4	Secondary malignant neoplasm of small intestine
C78.5	Secondary malignant neoplasm of large intestine and rectum
C78.6	Secondary malignant neoplasm of retroperitoneum and peritoneum
C78.7	Secondary malignant neoplasm of liver and intrahepatic bile duct
C78.8	Secondary malignant neoplasm of other and unspecified digestive organs
C78.80	Secondary malignant neoplasm of unspecified digestive organ
C78.89	Secondary malignant neoplasm of other digestive organs
C79.0	Secondary malignant neoplasm of kidney and renal pelvis
C79.00	Secondary malignant neoplasm of unspecified kidney and renal pelvis
C79.01	Secondary malignant neoplasm of right kidney and renal pelvis
C79.02	Secondary malignant neoplasm of left kidney and renal pelvis
C79.1	Secondary malignant neoplasm of bladder and other and unspecified urinary organs
C79.10	Secondary malignant neoplasm of unspecified urinary organs
C79.11	Secondary malignant neoplasm of bladder
C79.19	Secondary malignant neoplasm of other urinary organs
C79.2	Secondary malignant neoplasm of skin
C79.3	Secondary malignant neoplasm of brain and cerebral meninges
C79.31	Secondary malignant neoplasm of brain
C79.32	Secondary malignant neoplasm of cerebral meninges
C79.4	Secondary malignant neoplasm of other and unspecified parts of nervous system
C79.40	Secondary malignant neoplasm of unspecified part of nervous system
C79.49	Secondary malignant neoplasm of other parts of nervous system
C79.5	Secondary malignant neoplasm of bone and bone marrow
C79.51	Secondary malignant neoplasm of bone
C79.52	Secondary malignant neoplasm of bone marrow
C79.6	Secondary malignant neoplasm of ovary
C79.60	Secondary malignant neoplasm of unspecified ovary
C79.61	Secondary malignant neoplasm of right ovary
C79.62	Secondary malignant neoplasm of left ovary
C79.7	Secondary malignant neoplasm of adrenal gland
C79.70	Secondary malignant neoplasm of unspecified adrenal gland
C79.71	Secondary malignant neoplasm of right adrenal gland
C79.72	Secondary malignant neoplasm of left adrenal gland
C79.8	Secondary malignant neoplasm of other specified sites
C79.81	Secondary malignant neoplasm of breast
C79.82	Secondary malignant neoplasm of genital organs
C79.89	Secondary malignant neoplasm of other specified sites
C79.9	Secondary malignant neoplasm of unspecified site

**Table A2:** Comparison of comorbid condition incidence in the total sample and PCC group.

Comorbidities	All	PCC ^a^ (Yes)	
	N (%)	N (%)	*p*-Value
Sepsis	4924 (1.0)	1033 (3.6)	<0.0001
Weight Loss	43,385 (8.5)	7020 (24.0)	<0.0001
Acute Renal Failure	30,907 (6.0)	4999 (17.1)	<0.0001
Pneumonia	13,014 (2.5)	1737 (5.9)	<0.0001
Dehydration	33,306 (6.5)	3872 (13.2)	<0.0001
UTI ^b^	48,489 (9.5)	4017 (13.7)	<0.0001
Acute Cardiac Event	46,328 (9.0)	3648 (12.5)	<0.0001
Acute Pulmonary Event	24,358 (4.7)	4208 (14.4)	<0.0001
Acute Cerebrovascular Event	569 (0.1)	52 (0.2)	0.0005
Acute Hepatic Failure	2478 (0.5)	690 (2.4)	<0.0001

^a^ PCC, palliative care consultation. ^b^ UTI, urinary tract infection.
